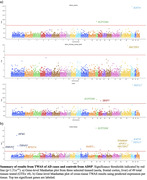# Known and novel genes significant to AD across tissues may help to uncover comprehensive changes of disease

**DOI:** 10.1002/alz.087088

**Published:** 2025-01-03

**Authors:** Anni Moore, Yuki Bradford, Rachit Kumar, Rasika Venkatesh, Manu Shivakumar, Jingxuan Bao, Li Shen, Dokyoon Kim, Marylyn D. Ritchie

**Affiliations:** ^1^ University of Pennsylvania, Philadelphia, PA USA

## Abstract

**Background:**

Alzheimer’s disease (AD) is neurodegenerative disease brought on by a combination of changes in multiple pathways that conglomerate to promote disease progression. AD often occurs alongside comorbid diseases, most often immune or vascular in nature, which have been shown to further increase AD risk. We previously showed that known AD variants also associate with secondary diseases in these categories, including rheumatoid arthritis, ischemic myocardial infarction, and both Type 1 and Type 2 diabetes. To better understand which pathways play a significant role in AD etiology and how comorbid diseases may contribute to risk through shared pathways, we aimed to identify gene expression changes associated with AD status in tissues throughout the body.

**Method:**

We conducted a transcriptome‐wide association study (TWAS) using genotypes from the Alzheimer’s Disease Sequencing Project (ADSP) including 11,074 cases and 14,310 controls from multiple ancestry groups and reference expression‐trait quantitative loci (eQTLs) from 49 tissues in Genotype‐Tissue Expression (GTEx) Project (v8). Using an ensemble approach we first predicted genetically regulated gene expression (GREx) and gene‐associations per tissue within each of the 49 tissues available in GTEx using PrediXcan. Secondly, to better predict cross‐tissue signals we used MulTiXcan with GREx to test for gene‐associations across tissues.

**Result:**

Both known and novel genes appeared in the single tissue and cross tissue gene associations. Previously AD‐associated genes *PET117*, *RAB35*, *NECTIN2*, *APOC1*, and *TOMM40* were significantly associated (p<1.51e‐7) with AD across tissues, with many of these known to lie within the *APOE* region. Additionally, genes previously unreported with AD, but involved in relevant disease processes including macro‐autophagy (*SUPT20H*), nucleosome binding (*HMGN2*), histone acetylation (*KAT14*), DNA damage repair (*HFM1*), and immune response (*TSPAN2*) showed significance. Many of these novel genes have previously been associated with immune, vascular and other neurological diseases, many of which are better known to appear as AD comorbidities.

**Conclusion:**

In addition to known *APOE* region genes, novel genes with implications relevant to AD etiology and ties to other immune, vascular, and neurological diseases also appeared significant across tissues. Next we will investigate how these gene expression changes apply to pathways within relevant tissues.